# Autoimmune encephalitis: what the radiologist needs to know

**DOI:** 10.1007/s00234-024-03318-x

**Published:** 2024-03-20

**Authors:** Francesco Sanvito, Anna Pichiecchio, Matteo Paoletti, Giacomo Rebella, Martina Resaz, Luana Benedetti, Federico Massa, Silvia Morbelli, Eduardo Caverzasi, Carlo Asteggiano, Pietro Businaro, Stefano Masciocchi, Lucio Castellan, Diego Franciotta, Matteo Gastaldi, Luca Roccatagliata

**Affiliations:** 1 Unit of Radiology, Department of Clinical, Surgical, Diagnostic, and Paediatric Sciences, University of Pavia, Viale Camillo Golgi, 19, 27100 Pavia, Italy; 2 UCLA Brain Tumor Imaging Laboratory (BTIL), Center for Computer Vision and Imaging Biomarkers, University of California Los Angeles, Los Angeles, CA USA; 3 Department of Radiological Sciences, David Geffen School of Medicine, University of California Los Angeles, Los Angeles, CA USA; 4 Department of Brain and Behavioral Sciences, University of Pavia, Pavia, Italy; 5 Advanced Imaging and Artificial Intelligence Center, Department of Neuroradiology, IRCCS Mondino Foundation, Via Mondino 2, 27100 Pavia, Italy; 6 IRCCS Ospedale Policlinico San Martino, Largo Rosanna Benzi 10, 16132 Genoa, Italy; 7 Department of Neuroscience, Rehabilitation, Ophthalmology, Genetics, Maternal and Child Health (DINOGMI), University of Genoa, Largo Daneo 3, 16132 Genoa, Italy; 8 Department of Health Sciences (DISSAL), University of Genoa, Via Antonio Pastore 1, 16132 Genoa, Italy; 9 Neuroimmunology Laboratory and Neuroimmunology Research Section, IRCCS Mondino Foundation, Via Mondino 2, 27100 Pavia, Italy

**Keywords:** Magnetic resonance imaging, Encephalitis, Limbic encephalitis, Autoimmune encephalitis, Autoimmune diseases of the nervous system

## Abstract

Autoimmune encephalitis is a relatively novel nosological entity characterized by an immune-mediated damage of the central nervous system. While originally described as a paraneoplastic inflammatory phenomenon affecting limbic structures, numerous instances of non-paraneoplastic pathogenesis, as well as extra-limbic involvement, have been characterized. Given the wide spectrum of insidious clinical presentations ranging from cognitive impairment to psychiatric symptoms or seizures, it is crucial to raise awareness about this disease category. In fact, an early diagnosis can be dramatically beneficial for the prognosis both to achieve an early therapeutic intervention and to detect a potential underlying malignancy. In this scenario, the radiologist can be the first to pose the hypothesis of autoimmune encephalitis and refer the patient to a comprehensive diagnostic work-up – including clinical, serological, and neurophysiological assessments.

In this article, we illustrate the main radiological characteristics of autoimmune encephalitis and its subtypes, including the typical limbic presentation, the features of extra-limbic involvement, and also peculiar imaging findings. In addition, we review the most relevant alternative diagnoses that should be considered, ranging from other encephalitides to neoplasms, vascular conditions, and post-seizure alterations. Finally, we discuss the most appropriate imaging diagnostic work-up, also proposing a suggested MRI protocol.

## Introduction

Autoimmune encephalitis (AE) refers to a spectrum of disorders characterized by inflammatory processes affecting the brain tissue and originating from an immune-mediated pathophysiological mechanism targeting neurons [1, 2]. The reported incidence ranges from 3/million to 8/million person-years in epidemiologic studies in the western world [3, 4], but many authors argue that this disease category is still under-diagnosed and misdiagnosed [5, 6].

AE was originally described as a paraneoplastic phenomenon affecting limbic structures, and causing subacute onset of behavior and memory disturbances, along with seizures, in the presence of an underlying neoplasms [7–9]. Subsequent evidence led to the detection of autoantibodies linked with AE [10, 11], and contributed to the recognition that non-paraneoplastic instances are relatively common [2]. Currently, AE includes a number of subtypes, that can be classified based on the associated autoantibody (Ab) found in the serum and/or CSF. AE Ab target either intracellular (Group I – such as anti-Hu, anti-Ma/Ta, anti-GAD, anti-Yo, and anti-CV2/CRMP5), or cell surface neuronal antigens (Group II – including anti-LGI1, anti-CASPR2, anti-GABA_A_R, anti-GABA_B_R, anti-NMDAR, and anti-AMPAR). This distinction bears significant clinical, prognostic, and pathophysiological implications, a general rule being that Group I AE are associated with a worse prognosis than Group II AE [1, 12]. Group I AE are more likely associated with an underlying malignancy (Group I Ab are also referred to as ‘onconeural antibodies’) [13]. For instance, anti-Hu AE – the most common paraneoplastic AE with a reported incidence of 0.4/million person-years [4] – is associated with tumors in 75–80% of cases (mainly small cell lung carcinoma, SCLC) [14, 15]. On the other hand, Group II AE are more commonly non-paraneoplastic conditions that can affect patients belonging to a wide age range, including younger and sometimes pediatric patients [2, 14]. Anti-NMDAR (N-methyl D-aspartate receptor) AE and anti-LGI1 (leucine-rich glioma inactivated) AE – the most common Group II AE, with a reported incidence of 0.5/million and 0.4/million person-years, respectively [4] – show an association with neoplasms in 30%-40% and in 10% of cases, respectively [14, 16]. Despite the abovementioned rule of thumb for neoplastic associations is overall valid, some AE subtypes represent notable exceptions. For instance, GABA_B_R (gamma-amino butyric acid B receptor) antibodies (group II) are frequently associated with SCLC, while GAD (glutamic acid decarboxylase) antibodies (targeting intracellular antigens) are typically associated with non-paraneoplastic AE, and they are more often associated with systemic autoimmune conditions (e.g. type 1 diabetes mellitus) rather than with neoplasms (< 10% of cases) [14]. More in general, different antibodies have a different likelihood of tumor associations, and the updated 2021 diagnostic criteria for AE [17] propose to stratify AE-related antibodies in low-, intermediate- and high-risk, based on the likelihood of tumor associations. In addition to a worse prognosis due to the underlying malignancies, Group I AE are generally characterized by a worse treatment response and are more likely to induce irreversible tissue damage [1]. Such differences are related to the underpinning pathophysiological mechanisms. While in Group II the autoantibodies are believed to serve a pathogenetic function, in Group I the immune-mediated damage is sustained by CD8 + T-cells and the extent of the antibody contribution to the neural damage is still being evaluated [13]. As a general rule, in Group I antitumoral immune response cross-reacts with neural antigens causing lymphocyte-mediated neuronal killing. Conversely, in Group II autoantibodies bind to cell-surface epitopes, typically belonging to ion-channels, and result in disease-causing synaptic function interference [1, 2, 16]. Therefore, in Group II AE antibody-depleting therapies (such as IVIG or plasma exchange/immunoadsorption) [16] are usually more effective and immune-mediated insults are more often reversible.

From a clinical perspective, most AE patients present with a subacute onset and progression (< 3 months) of various symptoms, mainly related to limbic disfunction, including memory deficits, psychiatric symptoms, seizures, and altered mental status – as reported in the diagnostic criteria for ‘possible AE’ [18]. When AE involves extratemporal structures presentations may also include movement disorders, ataxia, autonomic or sleep disorders, for instance. Although limbic encephalitis (LE) is one of the most common forms of AE, different AE subtypes are related to peculiar clinical findings. Anti-Hu AE often causes a sensory neuropathy and a cerebellar syndrome, in addition to or independently from the classic limbic involvement [15, 17]. While anti-GAD AE is usually a typical LE featuring prominent seizures, anti-Ma/Ta AE patients exhibit pure LE symptoms in a minority of cases, but often shows additional diencephalic or brainstem disfunction – e.g., gaze palsy [12, 19]. Anti-NMDAR AE causes the most defined clinical syndrome – leading to the proposal of specific and unique diagnostic criteria [18] – with a typical progression from viral-like prodromes to prominent psychiatric/behavioral symptoms, rapidly followed by memory deficits, language impairment, seizures, dyskinesias, altered state of consciousness and finally autonomic disfunction or central hypoventilation [16]. Anti-LGI1 AE also presents characteristic clinical features, such as faciobrachial dystonic seizures (FBDS), hyponatremia, and sleep disturbance [14, 20]. FBDS are briefs jerks involving in most cases the facial muscles associated with elevation and dystonic posturing of the ipsilateral upper limb (and more rarely the lower limb). These events can involve both sides and occur at a high frequency (even > 200 episodes/day). Anti-CASPR2 AE may have a wide clinical presentation, from pure limbic encephalitis, cerebellar dysfunction or peripheral nerve hyperexcitability to the well-defined Morvan syndrome where peripheral nerve hyperexcitability coexists with i) cognitive symptoms or seizures and ii) central autonomic disfunction or insomnia [14, 16, 21]. Anti-AMPAR (α-amino-3-hydroxy-5-methyl-4-isoxazolepropionic acid receptor) AE often shows a psychiatric syndrome in the context of a limbic encephalitis [12, 22]. When paraneoplastic, tumor types that are most commonly associated with AE include: SCLC (anti-Hu and multiple other Group I AE), ovarian tumors (anti-NMDAR and anti-Yo), testicular tumors (anti-Ma/Ta), Hodgkin lymphoma (Tr/DNER and multiple other Group II AE) [14, 16]. A more extensive overview of clinical syndromes and tumor types related to AE subtypes are provided in some recent articles [14, 16].

The Graus criteria [18] also allow for the diagnosis of AE in the *absence* of neuronal antibodies. Antibody-negative AE mostly presents with a limbic phenotype (seronegative definite LE) [23]. In addition, patients not fulfilling criteria for LE can be diagnosed in a specific subgroup named “antibody-negative but probable AE” (ANPRA), which remains heterogeneous and poorly defined [23]. Finally, a category named “possible AE” exists in the Graus criteria, but it should be intended as a “work-in-progress” category identifying patients that should undergo a thorough work-up for a definite diagnosis, including CSF testing. Overall, the existence of seronegative AE cases is due to a number of reasons, including some Ab not being detected by commercial assays (but exclusively by means of in-house assays in reference centers) [5], new AE-associated Ab being constantly discovered, and some cell-mediated AE potential lacking associated Ab [14, 24]. Challenges in diagnosis of antibody negative AE have been recently reviewed [25].

In this complex and constantly evolving scenario where the clinical onset may be insidious and the Ab detection is not always a decisive diagnostic tool, radiologists should be capable of recognizing the typical imaging features of limbic encephalitis (Section “Typical Limbic Encephalitis, imaging findings”) in order to pose the suspicion of AE in patients presenting with seizures, cognitive or psychiatric symptoms, and altered state of consciousness. In addition, the radiologist should be aware that AE may also present with peculiar features and/or extra-limbic involvement (Section “Imaging patterns of extra-limbic involvements”). The recognition of a potential AE is crucial, as it initiates a multidisciplinary effort – including accurate neurological evaluation, electrophysiological assessment, CSF and serum examination – aimed at a timely diagnosis. This is pivotal for prognosis, as it allows an early initiation of therapy and an immediate screening for potential underlying malignancies.

Furthermore, we provide an overview of the alternative diagnostic hypotheses (Section “Differential diagnosis”) – including infectious conditions, neoplasms, vascular and post-seizure alterations – that should be carefully ruled out since they require a different diagnostic and therapeutical management. Finally, in the light of the latest findings regarding AE radiological features, we suggest a dedicated imaging protocol for the diagnosis (Section “Suggested imaging diagnostic work-up”).

There are different approaches that can be used to classify AE [26]. These include a serological classification based on the type of antibodies, an etiological classification that distinguishes idiopathic AE, paraneoplastic AE, post infectious AE, and iatrogenic AE (associated for instance with immunomodulatory/suppressive drugs and immune check points inhibitors). Among the possible classification concepts, this review adopts an anatomical classification, the most useful for the radiologist. As a complementary approach, Tables 1 and 2 present a synopsis of clinical and radiologic findings associated with the main antibodies, also providing a brief overview of antibodies not discussed in detail in the text, along with the corresponding references. We purposefully did not include in this review MOG-IgG associated disorder (MOGAD) as, even though some of these patients can present with encephalitic manifestations, such as ADEM or cortical encephalitis, most patients present a demyelinating phenotype. Recently, radiological features of MOGAD have been reviewed elsewhere [27].

**Table 1:** Clinical and radiologic features of autoimmune encephalitides associated with autoantibodies targeting intracellular antigens

Abs target [reference]	Clinical syndrome	Ig subclasses	CSF findings	Tumor association	Neuroradiological pattern	Immunotherapy response
Hu (ANNA-1) [4, 15, 28]	Limbic encephalitis, cerebellar ataxia and/or sensory neuropathy	-	> 80% abnormal	75% lung cancer	10% abnormal; temporal T2 hyperintensity	Poor/absent
Yo (PCA-1) [17, 29, 30]	Subacute cerebellar ataxia	IgG1	> 90% abnormal	> 90% pelvic or breast tumor	Acute MRI mostly normal; cerebellar atrophy at follow-up	Poor/absent
CV2/CRMP5 [14, 31–33]	Striatal Encephalitis, cerebellar ataxia, myelopathy, chorea, peripheral and/or cranial neuropathy	-	> 90% abnormal	> 80% SCLC and thymoma	Striatal T2 hyperintensity	Poor/absent
Ma2 [14, 19, 33, 34]	Limbic, midbrain and/or brainstem encephalitis	-	80% abnormal	> 80% testicular tumors	70% abnormal; temporal and/or midbrain T2 hyperintensity with 50% contrast enhancement	30% improvement
GAD65 [14, 35, 36]	Limbic encephalitis, seizures, cerebellar ataxia and/or stiff person syndrome	-	30–100% abnormal	< 10% thymoma	Temporal T2 hyperintensity, 30% develop medial temporal sclerosis at follow-up	Highly variable, from no to complete response
Ri (ANNA-2) [37]	Cerebellar ataxia, isolated tremor and/or oculomotor alteration	-	> 95% abnormal	92% breast cancer	20% abnormal; brainstem T2 hyperintensity	Poor
Amphiphysin [38]	Myelopathy, Stiff-man syndrome, cerebellar ataxia, encephalopathy and/or limbic encephalitis	-	61% abnormal	79% lung and breast cancer	Normal or temporal T2 hyperintensities when limbic encephalitis	Poor
MAP1B (PCA-2) [39]	Peripheral neuropathy, myelopathy, cerebellar ataxia and/or cognitive decline/encephalopathy	-	74% abnormal	79% lung cancer	40–50% abnormal; spinal cord T2 hyperintensity with contrast enhancement, cerebral atrophy	58% improvement
SOX1 (AGNA) [40–42]	Lambert-Eaton myasthenic syndrome, cerebellar degeneration and/or encephalitis	-	> 60% abnormal	94% lung cancer	40–50% abnormal; temporal T2 hyperintensity, cerebellar atrophy	Variable but generally poor
KHLH 11 [43, 44]	Cerebellar ataxia and/or limbic encephalitis	-	100% abnormal	70% testicular seminoma	70% abnormal; midbrain/temporal/cerebellar T2 hyperintensity, mild cerebellar atrophy	69% improvement or stabilized
NIF [45]	Cerebellar ataxia, encephalopathy, myelopathy, limbic encephalitis and/or cranial neuropathies	-	70% abnormal	77% neuroendocrine tumors	40–50% abnormal; cerebellar atrophy, temporal T2 hyperintensity, cranial nerve enhancement)	70% improvement
PDE10A [46]	Movement disorders	-	100% abnormal	85%	80% abnormal; basal ganglia T2 hyperintensity, leptomeningeal enhancement	Poor
DACH1 (ANNA-3) [47]	Neuropathy, encephalitis and/or cerebellar ataxia	-	66% abnormal	90% neuroendocrine tumors	NA	70% improvement
Tr/DNER [48]	Subacute cerebellar ataxia and/or encephalopathy	IgG1 and IgG3	59% abnormal	89% Hodgkin lymphoma	Cerebellar atrophy	Poor
Trim46 [49, 50]	Subacute cerebellar ataxia, encephalopathy, movement disorder, limbic encephalitis, myelopathy and/or progressive dementia	-	64% abnormal	82% neuroendocrine tumors	50% abnormal; temporal T2 hyperintensity, cerebellar atrophy	Poor/absent
AP3B2 [51]	Cerebellar ataxia, spinocerebellar ataxia, myeloneuropathy and/or neuropathy	-	100% abnormal	10% renal cell carcinoma	75% abnormal; cerebellar/spinal cord atrophy	Stabilization, no improvement
RGS8 [52]	Cerebellar ataxia	IgG1	May be abnormal	lymphoma	At follow-up cerebellar atrophy	Variable, possible improvement
AK5 [53]	Limbic encephalitis, rapidly progressive amnesia	> IgG1	100% abnormal	< 5%	90% abnormal at onset; temporal T2 hyperintensity, 90% abnormal at follow-up; severe atrophy	20% improvement
AGO [54]	Limbic encephalitis, sensory neuronopathy and/or cerebellar ataxia	> IgG1	50% abnormal	20% miscellaneous	> 90 abnormal; temporal T2/FLAIR hyperintensity, cerebellar atrophy	60% improvement, 20% stabilization
GFAP [55]	Encephalitis, meningitis, myelitis and/or papillitis	-	> 80% abnormal	30% ovarian teratoma in presence of concomitant NSAbs	> 60% abnormal; brain linear perivascular radial enhancement	> 80 improvement
Zic4 [56, 57]	Subacute cerebellar ataxia	-	May be normal	90% lung cancer	Cerebellar atrophy	Poor
Septin 5 and 7 [58]	Cerebellar ataxia, encephalopathy, eye movement alteration and/or myelopathy	IgG1 and IgG2	73% abnormal	15%	30% abnormal in Septin-5; cerebellar atrophy - 60–70% abnormal in Septin-7; temporal T2/FLAIR hyperintensity, cerebral atrophy	80% improvement

ANNA: anti-neuronal nuclear antibody, PCA: anti-Purkinje cell cytoplasmic antibody, CRMP5: collapsin response mediator protein 5, GAD: glutamic acid decarboxylase, MAP1B: microtubule-associated protein 1B, SOX: Sry-like high mobility group box, AGNA: anti-glial nuclear antibody, KLHL11: Kelch-like protein 11, NIF: neuronal intermediate filament, PDE: phosphodiesterase, DACH1: Dachshund-homolog, DNER: Delta/Notch-like epidermal growth factor-related receptor, Trim: tripartite motif-containing protein, AP3B2: AP-3 complex subunit beta-2, RGS: regulator of G-protein signaling, AK: adenylate kinase, AGO: Argonaute, GFAP: glial fibrillary acidic protein, Zic: Zinc-finger protein

**Table 2:** Clinical and radiologic features of autoimmune encephalitides associated with autoantibodies targeting neuronal surface antigens

Abs target [reference]	Clinical syndrome	Ig subclasses	CSF findings	Tumor association	Neuroradiological pattern	Immunotherapy response
NMDAR [59–62]	Encephalitis, neuropsychiatric manifestations, movement disorders, dysautonomia and/or coma	IgG1	80% abnormal	> 40% ovarian teratoma	Mostly normal. In a minority temporal lobe T2 hyperintensity	80% improvement; a second line is frequently required
LGI1 [20, 63]	Limbic encephalitis, facio-brachial dystonic seizures and/or hyponatremia	> IgG4 but also IgG1-IgG2	25% abnormal	< 5% thymoma	70% abnormal; temporal T2 hyperintensity, less commonly basal ganglia T2/FLAIR hyperintensity	60–70% improvement
CASPR2 [21, 64]	Limbic encephalitis, dysautonomia, insomnia, peripheral nerve hyperexcitability and/or neuropathic pain	> IgG4 but also IgG1	35% abnormal	15% thymoma	30% abnormal; temporal T2 hyperintensity	> 80% improvement
GABA_A_ R [65–67]	Encephalitis, status epilepticus, behavior alteration and/or movement disorders	IgG1	60% abnormal	25% thymoma	90% abnormal; multifocal temporal and frontal T2 hyperintensity (GABAA R pattern)	> 80% improvement
GABA_B_ R [68, 69]	Limbic encephalitis	IgG1	> 90% abnormal	50% small cell lung cancer	60% abnormal; temporal T2 hyperintensity	75% improvement
AMPA R [22, 70]	Limbic encephalitis, memory loss, psychosis and/or motor deficits	IgG1	70% abnormal	65% lung cancer and thymoma	70% abnormal; temporal T2 hyperintensity, less commonly multifocal T2/FLAIR hyperintensity	70% improvement
Neurexin-3α [71]	Encephalitis, decreased level of consciousness and/or movement disorders	-	> 90% abnormal	< 5%	20% abnormal; temporal T2 hyperintensity	60% improvement
IgLON5 [72, 73]	Brainstem encephalitis, obstructive sleep apnoea and/or chorea. ALS like syndrome	> IgG4 but also IgG1	60% abnormal	< 5%	> 90% normal	50% improvement but not sustained
DPPX [74–76]	Encephalitis with prodromal weigh loss or diarrhea followed by cognitive dysfunction, CNS hyperexcitability, cerebellar and/or brainstem dysfunction	IgG1 and IgG4	50–60% abnormal	< 5%	> 90% normal or aspecific findings	60% improvement
D2R [77]	Basal ganglia encephalitis and/or psychiatric dysfunction	NA	75% abnormal	< 5%	50% abnormal; basal ganglia T2 hyperintensity	Apparently good
mGluR5 [78]	Encephalitis, psychiatric and cognitive symptoms, movement disorders, sleep dysfunction	IgG1 but also IgG2-IgG3	> 90% abnormal	50% Hodgkin lymphoma	50% abnormal; extralimbic regions T2 hyperintensity	> 80% improvement
mGluR1 [79]	Cerebellar ataxia and behavioural/cognitive symptoms	IgG1	> 80% abnormal	10% lymphoma	40% abnormal; cerebellum T2 hyperintensity, less commonly subcortical lesions/leptomeningeal enhancement, 80% cerebellar atrophy at follow-up	40% improvement
GluK2 [80]	Cerebellar ataxia	IgG1	> 90% abnormal	Rare but high in presence of concomitant NSAbs	> 75% abnormal; cerebellum T2 hyperintensity or atrophy	50–60% improvement
SEZ6L2 [81]	Cerebellar ataxia and mild extrapyramidal symptoms	IgG4	25% abnormal	< 5%	> 90% normal	Poor

NMDAR: N-methyl D-aspartate receptor, LGI: leucine-rich glioma inactivated, CASPR2: contactin-associated protein-like 2, GABAR: gamma-amino butyric acid receptor, AMPAR: α-amino-3-hydroxy-5-methyl-4-isoxazolepropionic acid receptor, IgLON5: immunoglobulin-like cell adhesion molecule 5, DPPX: dipeptidyl aminopeptidase-like protein, D2R: dopamine receptor 2, mGluR: metabotropic glutamate receptor, GluK2: glutamate kainate receptor subunit 2, seizure-related 6 homolog like 2

## Typical Limbic Encephalitis, imaging findings

Limbic encephalitis (LE) represents the typical presentation of AE, and was first described in the 1960s as a clinico-pathological entity [7, 82] with medial temporal lobe (MTL) symptoms caused by an inflammatory process involving structures of the limbic system, including the hippocampus, amygdala, hypothalamus, cingulate gyrus and limbic cortex. Reports on the MRI correlates of this neuropathology were published at the end of the 1980s [83]. Although imaging findings are often not confined to these areas, the identification of bilateral involvement of the MTL on T2-weighted MRI is a key diagnostic feature in the typical pattern of LE and this MRI pattern is one of the four diagnostic criteria which are necessary for a diagnosis of definite LE according to Graus et al. [18]. Importantly, this MRI finding enables a diagnosis of definite AE in the pertinent clinical scenario even in the absence of neuronal antibodies. Conversely, in the presence of a negative MRI, unilateral medial temporal lobe anomalies or other MRI patterns (cortical/subcortical, striatal, diencephalic, brainstem, encephalomyelitis, and meningoencephalitis) only a diagnosis of possible or probable AE can be formulated unless there is evidence of neuronal antibodies. The role of MRI is obviously also to rule out other non-immune disorders that may have unilateral involvement such as seizures, herpes simplex virus encephalitis or gliomas. FDG-PET can be useful in identifying metabolic alterations in temporo-medial regions when the MRI is negative and thus serves as a substitute of the MRI abnormalities to fulfill diagnostic criteria for definite LE [18].

### AE subtypes

The MRI pattern of anatomical involvement of the limbic lobe has been associated with numerous auto-antibodies, and, despite a certain variability across-studies, some AE subtypes seem to more likely present with LE. Anti-LGI1 AE, among the most frequent types of AE overall, is also considered one the most frequent cause of non-paraneoplastic LE [84, 85] – although a non-negligible portion of anti-LGI1 AE cases is paraneoplastic, and specifically associated with thymomas [86]. Across studies, anti-LGI1 AE is reported to show the typical LE pattern in 73–83% of the cases [63, 87]. For instance, in the cohort described by van Sonderen and colleagues [88], LE was present in 34 out of 38 patients, and the typical hippocampal T2-hyperintensity was observed in 79% of the patients. In another study on 76 patients with anti-LGI1 associated cognitive impairment, Ariño et al. [63] found a typical LE pattern in 83% of cases, while the remaining cases showed either non-LE (4%) or encephalopathy (13%, with no MRI and CSF anomalies). Along with anti-LGI1, anti-CASPR2 (contactin-associated protein-like 2) antibodies are part of the voltage-gated potassium channel-complex (VGKC) antibodies. For anti-CASPR2 AE, despite LE is considered a common presentation [89], typical MRI findings are reported to be less frequent. In a cohort of 33 patients van Sonderen et al. [21] reported medial temporal lobe T2-hyperintensity in only 8 subjects (24%) – bilateral in all cases. Binks and colleagues [90] reported up to 50% of cases of anti-LGI1 and anti-CASPR2 being MRI-negative, therefore a potential diagnosis of these AE subtypes should not be ruled out in the absence of suspicious MRI findings. Other AE subtypes consistently associated with typical medial temporal lobe MRI alterations include anti-GABA_B_R AE (ranging ~ 50–60% of cases across studies) [68, 91] and anti-AMPAR AE (~ 55% of cases) [22]. Anti-GAD antibodies, too, tend to present as a typical LE when causing encephalitis (reportedly, in 59% of cases) [92]. Other AE subtypes, such as anti-Hu and anti-Ma/Ta are not exclusively associated with LE findings [12], while anti-CV2/CRMP5 (collapsin response mediator protein 5) AE are classically extra-limbic and present as striatal encephalitis (see Section “Imaging patterns of extra-limbic involvement”), rarely showing temporal involvement [85]. Finally, it is worth remembering that MRI can be unremarkable in patients with clinical features of LE. Among MRI-negative AE, it is worth mentioning anti-NMDAR AE. While not strictly considered LE, NMDAR can also present with limbic symptoms, and very frequently MRI negative (in ~ 70–90% of cases across studies) [61, 93, 94]. Indeed, for the dedicated 2016 diagnostic criteria for NMDAR-AE [18], MRI findings are not taken into consideration.

### T2/FLAIR findings

LE is characterized by a typical MTL involvement that can be evaluated on T2-weighted and T2-weighted FLAIR images. For brevity, T2-weighted images will be referred to as “T2” and T2-weighted FLAIR images will be referred to as “FLAIR”, while “T2/FLAIR” will refer to findings that are appreciable on T2 and/or FLAIR. The hallmarks of MTL AE are T2/FLAIR hyperintensity and swelling of the hippocampi and amygdalae. In 2006, based on MRI findings from a historic case series, Urbach et al. [95] summarized the typical essential features and progression pattern of LE as follows: unilateral or bilateral hyperintensity and swelling of MTL structures within ~ 3 months from the clinical onset, regression of the swelling within ~ 9 months, and gradual volume loss (i.e. atrophy) starting within ~ 1 year. However, it is to note that, even in their cohort, the timing of these findings was highly variable across patients, with some cases of absent swelling, swelling persisting for over one year, or atrophy appearing within few months. In a more recent study enrolling patients with LE [96], quantitative volumetric analyses of MTL demonstrated a bilateral volume increase in amygdala at baseline, followed by a regression of the swelling at the follow-up scan (~ 6 months), and by a volume loss due to atrophy at the third scan (~ 1 year). Notably, as opposed to anti-GAD AE, in anti-VGKC AE the volume alterations also involved the hippocampus, and were more pronounced. Overall, MTL swelling is reported to occur in 63–78% of anti-VGKC AE [97, 98]. Further evidence demonstrated that the volume increase of MTL structures is more prominent in the early phases of the disease, that may be asymmetric, and that different AE subtypes may present specific volumetric alteration patterns [99].

Even though LE is unilateral in a number of cases (up to ~ 50%, according to a recent case series [98], many authors agree that the typical presentation is with a bilateral involvement of MTL, sometimes asymmetric [100]. Conversely, in the presence of unilateral MTL alterations, the hypothesis of Herpes Simplex Encephalitis (HSE) should be carefully taken into consideration [100]. Consistently, according to the 2016 criteria, in the absence of positive neuronal antibodies bilateral MTL involvement is a necessary finding for the diagnosis of “definite” LE [18].

**Fig. 1:**
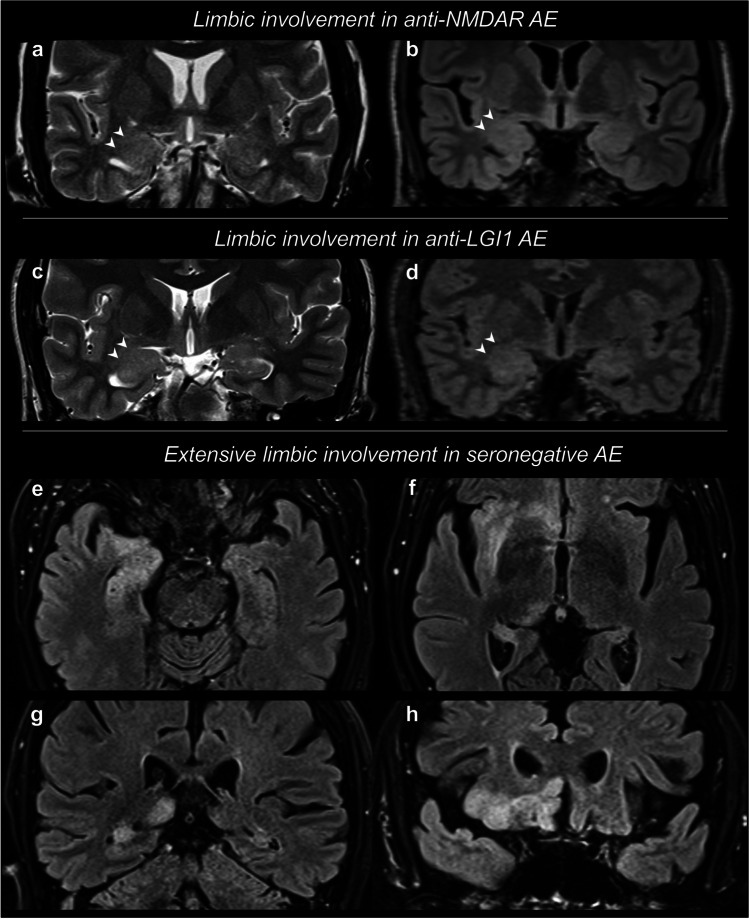
Limbic encephalitis. Limbic encephalitis (LE) can present with subtle radiologic findings, such as a swelling of the amygdala, with associated subtle signal hyperintensity, as visible (arrowheads) on coronal T2-weighted (**a**, **c**) and T2-weighted FLAIR (**b**, **d**) in two distinct cases of anti-NMDAR AE (**a**, **b**) and anti-LGI1 AE (**c**, **d**). Other patients may demonstrate more extensive alterations of the limbic structures. In this case (**e**–**h**), LE extensively involved the right limbic system, including amygdala (**e**), head of the hippocampus (**e**), body and tail of the hippocampus (**e**, **f**, **g**), insular cortex (**f**), thalamus/pulvinar (**f**), basal frontal gyri (**f**, **h**), and cingulate gyrus (**h**). White matter alterations are also noted, involving the external and extreme capsule (**f**). Following a thorough diagnostic work-up, a diagnosis of seronegative AE was posed

Regarding T2/FLAIR alterations, it is worth mentioning that the identification of subtle signal and volume changes in MTL structures may be challenging and reader-dependent, especially in patients with a mild disease. Figure 1a–d shows two cases of LE with subtle radiologic findings. For instance, in a recent study by Schievelkamp and colleagues [101], the authors reported an unsatisfactory diagnostic accuracy of the readers in distinguishing LE patients from age-matched healthy controls (accuracy: 64–74%), and a rather low inter-reader agreement for the identification of MTL signal and volume abnormalities. To address this issue, some authors proposed automated quantitative approaches to objectively identify MTL T2-signal alterations and aid the diagnosis of LE [102].

In association with MTL involvement, the typical LE pattern sometimes shows a more wide-spread limbic involvement, with a T2-signal abnormality and grey matter swelling also in the insula, in the lateral aspects of the temporal lobe, in the basal aspects of the frontal lobe, and in the cingulate gyrus; in addition, basal ganglia and thalamus involvement is not infrequent [100, 103]. Figure 1e–h displays a case of LE with extensive involvement of the limbic system structures.

Hypointense foci on T2*-weighted images, consistent with the presence of blood degradation products, are rare in AE [104].

### Contrast Enhancement (CE) and Diffusion Weighted Imaging (DWI)

CE and/or DWI restriction in the involved brain regions can occur in some cases, but is not considered typical of LE [18, 105]. On the contrary, prominent CE and a clear DWI restriction are often considered hallmarks of HSE [105, 106].

**Fig. 2:**
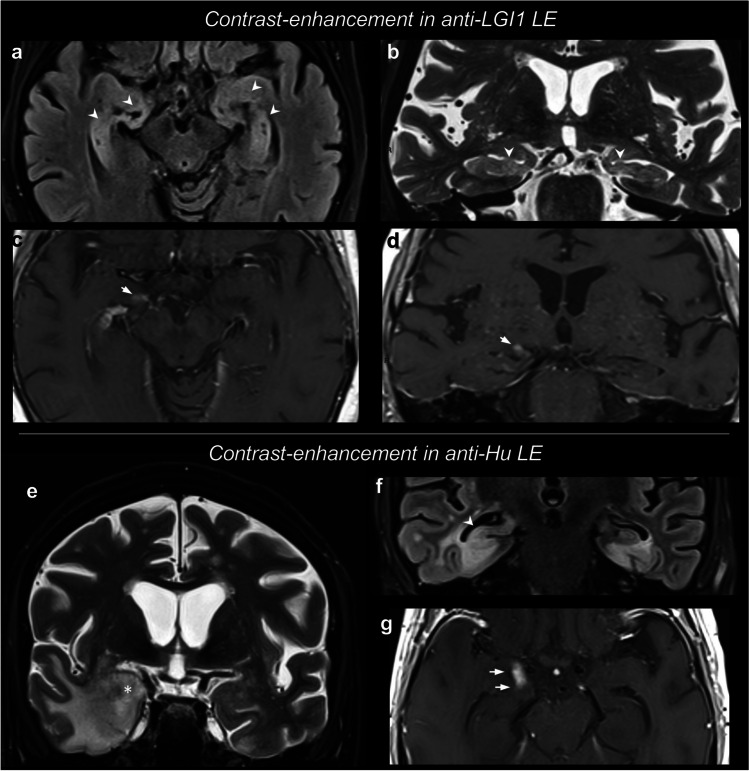
Limbic encephalitis with contrast-enhancement. Both cases presented with an encephalitic bilateral involvement of the medial temporal lobe (MTL) structures, and unilateral areas of contrast-enhancement in the right amygdala (arrows). The arrowheads show the bilateral areas of T2/FLAIR hyperintensity in the MTL. The anti-LGI1 AE case shows more symmetric involvement of the amygdalae and hippocampi on T2/FLAIR. The anti-Hu AE case shows an asymmetric pattern, with a more predominant swelling and hyperintensity of the right amygdala (asterisk in e) and hyperintensity of the right body of the hippocampus (arrowheads in f). These alterations are associated with MTL grey and white matter hyperintensity, including edema with finger-like appearance

In a recent case series, Kotsenas and colleagues [98] described a mild MTL CE in 28% of cases, and MTL DWI restriction in 43% of cases. Notably, in this cohort, CE and DWI restriction were significantly associated with the development of mesial temporal sclerosis (MTS) in the follow-up scans. Since CE was also previously reported in 15–25% of cases of LE [107], the presence of mild, patchy or poorly-delimited enhancing areas can be considered as a possible finding in a minority of LE cases. A peculiar instance is represented by anti-Ma AE, for which CE is reportedly more frequent (up to ~ 38%) [19]. Figure 2 shows two cases of LE with contrast-enhancement, both with bilateral yet asymmetric findings.

**Fig. 3:**
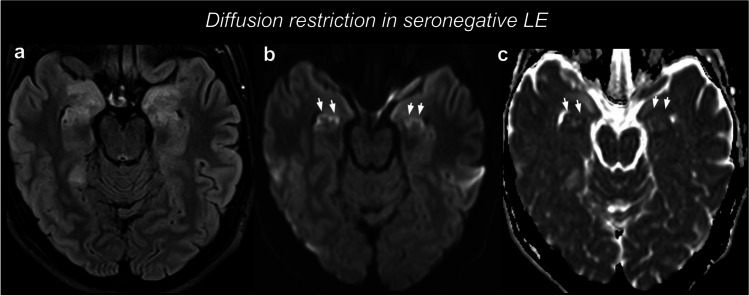
Limbic encephalitis with diffusion restriction. In this seronegative case, T2-weighted FLAIR images (**a**) showed a marked symmetric swelling and hyperintensity of the amygdala and head of the hippocampus. DWI (**b**) and ADC (**c**) revealed a gyriform diffusion restriction in the hippocampus (arrows), with ADC values as low as 0.58 × 10^3^ mm^2^/s (right) and 0.72 × 10^3^ mm^2^/s (left). After a diagnostic work-up, a diagnosis of seronegative AE was posed

Conversely, the occurrence of DWI restriction is more controversial. In other cohorts of LE, MTL lesions showed either subtle DWI changes only in few cases [108], or no DWI changes in any cases [109]. In addition, many articles reporting DWI changes [98, 100] did not evaluate ADC maps, and a recent study demonstrated that anti-LGI1 AE is characterized by DWI hyperintensity without ADC decrease [103]. Therefore, in many cases DWI changes may be ascribable to the “shine-through” effect, which was extensively described for AE [12, 110]. In fact, in a recent paper, only 9% of patients with LE showed reduced ADC values demonstrating an actual DWI restriction [111]. Overall, we can state that in AE cortical involvement with restricted diffusion on DWI is uncommon unless related to cytotoxic edema associated with epileptic activity. Outside this instance, cortical involvement with restricted diffusion should lead to consider other pathological entities in the differential diagnosis, for instance prion diseases as described in the section of the differential diagnosis. However, diffusion restriction can be seen in AE, and Fig. 3 represents an example of LE with symmetric and bilateral diffusion restriction.

### Atrophy and Mesial Temporal Sclerosis (MTS)

As already mentioned, LE may result in MTL volume loss due to atrophy in follow-up scans ~ 1 year after the disease onset, even though atrophy appears earlier in some cases [95, 96]. In a subset of patients, such atrophy displays the characteristic imaging features of MTS. MTS, also referred to as hippocampal sclerosis, consists in chronic gliosis and neural loss. On T2/FLAIR images, MTS can be identified as a signal increase and volume loss of hippocampus [112]. The degree of atrophy can be assessed on coronal reconstructions of 3D T1-weighted imaging that highlight a reduction in volume of the hippocampus and amygdala with relative dimensional increase of the temporal horn of the lateral ventricle and choroidal fissure. The hyperintensity is usually easier to visualize on T2/FLAIR images, but needs to be confirmed with T2 weighted images, that are considered more reliable and less prone to false positive findings [112]. While historically considered as both a cause and a consequence of temporal lobe epilepsy [113], the radiological appearance of MTS has been more recently identified as a possible sequela of encephalitis [12, 114, 115]. The prevalence of MTS as a sequela of LE is reported to be ~ 43–50% in anti-LGI1 AE cases across series [88, 98, 103], and ~ 33% in anti-GAD AE [36]. Notably, in a series including anti-VGKC AE patients, anti-LGI1 but not anti-CASPR AE evolved in MTS and DWI changes and CE at baseline predicted MTS [98].

**Fig. 4:**
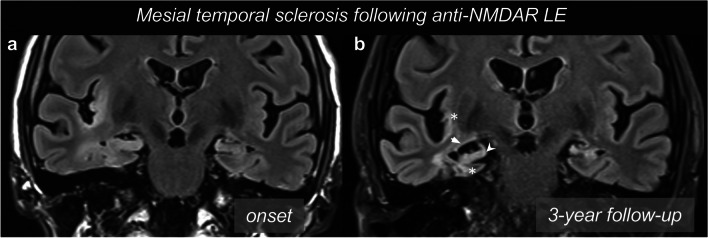
Medial temporal sclerosis as a sequela of limbic encephalitis. In this case of anti-NMDAR AE, T2-weighted FLAIR images (**a**) exhibited signal hyperintensity in the right MTL structures and insular cortex. The encephalitic insult evolved with atrophic changes over time. After three years, T2-weighted FLAIR images (**b**) showed medial temporal sclerosis, with volume loss of amygdala and hippocampus (arrowhead), as well as atrophy of the parahippocampus and insular cortex (asterisks), associated with an enlargement of the temporal horn of the lateral ventricle and choroidal fissure (arrow)

Interestingly, evidence from the literature suggests that hippocampal volumetric and morphological anomalies after AE may be characterized by peculiar features. A recent article [36] applied advanced volumetric and shape analyses and demonstrated that chronic alterations in anti-GAD AE consist in subtle shape abnormalities of selected areas in the hippocampus head, rather than a clear hippocampal atrophy, while other areas (including CA3 and hippocampal fissure) seem to be spared by the atrophy. Conversely, a cohort of patients with non-immune temporal lobe epilepsy presented a more widespread volume loss and deformation of the hippocampi, as well as a higher occurrence of hippocampal sclerosis. Figure 4 features images of a LE which resulted in mesial temporal sclerosis.

## Imaging patterns of extra-limbic involvement

Aside from the typical limbic pattern, AE can involve different extra-limbic anatomical structures. For the purpose of this review, we have grouped AE which may present with extra-limbic involvement in: cortical AE, perivascular involvement, striatal AE, diencephalic AE, AE with involvement of the rhombencephalon (brainstem and/or cerebellum). Combined involvement of different structures can be seen, including combined limbic and extra-limbic patterns. Of note, extra-limbic PET alterations both in terms of hypo- and hypermetabolism have been reported for AE [116].

### Cortical involvement

Anatomical involvement of cortex can be seen mainly in anti-NMDAR, anti-MOG (myelin oligodendrocyte glycoprotein), and anti-GABA_A_R AE. In anti-NMDAR AE Zhan et al. found MRI abnormalities in 49% of patients, 72% of which showed involvement of cortex of different lobes, with or without a combined hippocampal involvement [117]. Antibodies to the glial protein MOG can be associated with acute disseminated encephalomyelitis (more frequent in the pediatric population) and cerebral cortical encephalitis (more frequent in adults), and, as such, have been included into the spectrum of AE [17]. The cortical encephalitis is characterized by a unilateral [118] or bilateral frontal cortex involvement [119]. In these patients, who typically present with focal seizures, often evolving to bilateral tonic–clonic seizures, brain MRI demonstrates unilateral or bilateral cerebral cortical hyperintensities on T2/FLAIR sequences, with swelling of the cortex. Anti GABA_A_R AE affects mostly children and young adults, presenting most commonly with seizures (88%), cognitive impairment (67%), behavioral changes (46%), alterations of consciousness (42%), or abnormal movements (35%). T2/FLAIR MRI can demonstrate multifocal bilateral or unilateral cortical and subcortical areas of hyperintensity predominantly occurring in the temporal and frontal lobes, and less frequently in the parietal or occipital lobes. Basal ganglia and cerebellum can also be involved [66]. Interestingly, these multifocal T2/FLAIR changes can be asynchronous, with some appearing while others are disappearing along the disease course. On post-contrast T1-weighted images these lesions do not show enhancement although cases with gyriform leptomeningeal enhancement have been described. Typically, lesions are not characterized by restricted diffusion on DWI.

### Perivascular involvement

**Fig. 5:**
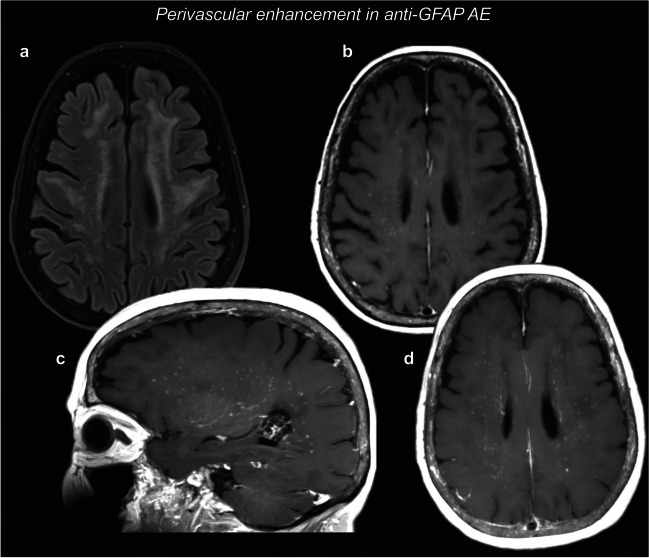
Perivascular enhancement in the centrum semiovale. T2-weighted FLAIR images (**a**) showed widespread signal alterations in the bi-hemispheric white matter. Post-contrast T1-weighted images (**b**) showed numerous spots of perivascular enhancement in the centrum semiovale, which can be more easily appreciated with maximum intensity projection (MIPs) images (**c**, **d**). Anti-GFAP autoantibodies were positive in CSF and serum

A very peculiar case is represented by anti-GFAP (glial fibrillary acidic protein) AE. The most distinctive finding is a perivascular radial contrast-enhancement in the centrum semiovale, perpendicular to the lateral ventricles, which can be found in ~ 50% of the cases, and can co-localize with white matter T2/FLAIR hyperintense areas [55]. These findings are clearly displayed in the representative case in Fig. 5. Alternatively, this radial pattern can be seen in the cerebellum in a minority of cases. Other MRI abnormalities include leptomeningeal or ependymal enhancement, serpentine enhancement, and/or accompanying long-segment spinal cord alterations [55, 120]. Given the characteristic radiologic appearance of radial contrast-enhancement in anti-GFAP AE, the radiologist is sometimes the first physician to pose the suspicion of this type of encephalitis.

### Basal ganglia involvement

Anatomical involvement of basal ganglia can be seen in AE with anti-CV2/CRMP5, anti-D2R (dopamine receptor 2), anti-NMDAR, and sometimes anti-LGI1 Ab. Anti-CV2/CRMP5 IgG target an intracellular antigen (collapsin response mediator protein) and have been reported in the setting of various paraneoplastic syndromes, including peripheral neuropathy, cranial neuropathy, gastroparesis, encephalitis, cerebellar ataxia, myelopathy, and chorea. Patients with anti-CV2/CRMP5 present with chorea or involuntary movements, and striatal involvement on MRI T2-weighted sequences, without diffusion restriction. Striatal involvement associated with spinal cord signal alterations has been reported [31]. Anti-CV2/CRMP5 has a strong cancer association, in particular with small-cell lung cancer or thymoma [121]. Another antibody recently described in patients with renal cancer and lung cancer is phosphodiesterase 10A IgG (PDE10A). Half of the patients described by Zekeridou et al. [46] had chorea or ballismus. It is interesting to note that in two of these patients the onset of movement disorders was described after the use of immune checkpoint inhibitor. MRI showed T2/FLAIR hyperintensities in the basal ganglia [46]. Differential diagnosis should include more common toxic and metabolic disorders with basal ganglia involvement and lack of restricted diffusion is helpful in the differential diagnosis with Creutzfeldt-Jakob disease (CJD). There are sporadic cases of anti-NMDAR AE with basal ganglia involvement. In some reported pediatric cases, basal ganglia involvement was characterized by restricted diffusion on DWI [122, 123] and, exceptionally, also a thalamic involvement was described [17]. In the context of anti-LGI1 AE some patients with faciobrachial dystonic seizures may show T1 and/or T2 hyperintensity (alone or combined) in the basal ganglia, with T1 hyperintensities persisting longer than the T2 hyperintensities (median 11 weeks vs 1 week) [124].

### Diencephalic involvement

In AE diencephalic-hypothalamic dysfunction can present with endocrine abnormalities, hyperthermia, hyperphagia, somnolence but also other severe dysautonomia symptoms. MRI T2/FLAIR hyperintensities can be detected in thalamus, geniculate bodies, hypothalamus and subthalamic nuclei, usually with bilateral and relatively symmetric involvement. In anti-Ma2 AE diencephalic involvement is usually seen in combination with abnormalities in the limbic system, although isolated diencephalic involvement has been reported [34]. Enhancement can be seen in approximately half of the patients in at least one of the areas abnormal on T2/FLAIR [19].

### Brainstem involvement

In rhombencephalitis the inflammatory process involves the brainstem with variable involvement of the cerebellum. In the acute phase MRI may be unremarkable or demonstrate T2/FLAIR hyperintensity within the brainstem, with or without cerebellar abnormalities. In the chronic phase parenchymal volume loss may ensue. Various antibodies are associated with brainstem involvement, the most common being anti-Ma2, anti-Ri, and anti-KLHL11 (Kelch-like protein 11). Patients with anti-Ma2 AE and brainstem encephalitis most often have ophthalmoplegia [19]. In anti-Ma2 AE encephalitis MRI is abnormal in up to 74% of cases. Limbic encephalitis is the most common pattern, but this AE may present with different combinations of limbic, diencephalic, or brain stem encephalitis. Brainstem encephalitis at MRI is more often associated with lesions in the midbrain and less commonly in the pons and/or medulla oblungata. Anti-Ri AE was initially described in association with opsoclonus–myoclonus syndrome and cerebellar ataxia in women with breast cancer but it can present with a wider spectrum of neurologic involvement, the cerebellum and the brainstem being most commonly affected. Brain MRI has been reported as pathological in 18% of cases, most commonly with T2 signal changes in the brainstem [37]. In anti-Hu AE Dalmau et al. [28] reported occurrence of symptoms associated with brainstem dysfunction in 31%. MR imaging findings usually correlate with clinical features and typically include T2/FLAIR hyperintense lesions in the medial temporal lobes with variable involvement of the cerebellum and brain stem. It is noteworthy that MRI detectable lesions reflecting clinical features in brainstem syndromes are frequently absent. In a series of 14 patients with anti-Hu associated brainstem encephalitis MRI was always normal [125]. Anti-KLHL11 encephalitis is a relatively recent pathological entity, most commonly presenting with a rhombencephalitis phenotype with ataxia, diplopia, dysarthria, vertigo, hearing loss, and tinnitus. A recent study described a strong association with testicular tumors [126]. In this cohort [126], MRI has been reported as abnormal in 76% (n = 28), showing T2/FLAIR hyperintensity, which were most commonly seen in the temporal lobe (n = 12), followed by cerebellum (n = 9), and more rarely in the brainstem (n = 3) and diencephalon (n = 3). In one patient there was also spinal cord central gray matter involvement. In a few instances there was associated enhancement (n = 3) and in single case leptomeningeal and cranial nerve V enhancement. At follow-up, cerebellar atrophy or medial temporal lobe atrophy was reported. Interestingly, three patients had hypertrophic olivary degeneration.

### Cerebellar involvement

Cerebellar ataxia has been described as a typical feature in the setting of paraneoplastic syndromes, and onconeural Ab positivity has been reported in a large number of patients with paraneoplastic cerebellar degeneration. Some of the antibodies most commonly associated with cerebellar involvement are anti-Yo/PCA11 (38%) and anti-Hu/ANNA-1 (32%) [28]. The ataxic syndrome associated with anti‐Yo antibody, or anti-PCA1 (Purkinje cell cytoplasmic antibody type 1), is the most common among the forms of paraneoplastic cerebellar degeneration (PCD). It typically presents with subacute development of pancerebellar deficits reaching clinical plateau within 6 months. The majority of cases have been reported in women in association with pelvic or breast tumors. There can be clinical manifestations of cerebellar dysfunction also in anti-Ma and anti-CV2/CRMP5 AE, and in anti-NMDAR AE cerebellar symptoms have been described in the pediatric population, while rare in adults [127–129]. In these cases of autoimmune cerebellitis, MRI is often unremarkable at presentation, even though T2/FLAIR hyperintensity of cerebellar hemispheres can be seen, while a more typical imaging finding is paraneoplastic cerebellar degeneration (PCD), consisting in cerebellar atrophy at follow-up, manifesting months to years later [29]. Finally, it is worth mentioning anti-GluK2 (glutamate kainate receptor subunit 2) AE, which can cause cerebellitis and can acknowledge obstructive hydrocephalus as a complication [80].

## Differential Diagnosis

Depending on anatomical sites involved and MRI signal features, AE can present with radiological characteristics resembling other diseases, including other encephalitides, neoplasms, vascular conditions, and post-seizure alterations. A correct differential diagnosis is crucial for patient management, in particular to promptly initiate an effective therapeutic strategy.

### Herpes Simplex Encephalitis (HSE)

HSE is the most important alternative diagnosis to consider when suspecting limbic encephalitis, since both entities tend to involve the mesial temporal structures and HSE is rather common, accounting for ~ 20% of limbic encephalitides overall [85]. Both clinical and imaging presentations can aid distinguishing between these two conditions.

From a clinical standpoint, HSE is more prone to present abruptly and with fever [85, 130], and is more typically associated with abnormal CSF findings [85]. Conversely, psychiatric manifestations point towards AE [131]. For instance, in a study comparing HSE and AE patients, psychiatric symptoms were exclusively seen in AE [100]. Additional clinical features more frequent in AE include memory deficits, seizures, and involuntary movements [132].

The limbic system involvement on MRI is more typically bilateral in AE, whereas in HSE is generally unilateral (or bilateral and asymmetric in some cases) [100]. In addition, the presence of striatal or thalamic MRI abnormalities advocates for a diagnosis of AE [85, 132]. In a recent study enrolling 95 patients with infectious or autoimmune encephalitis [132], the hippocampal involvement was significantly more frequent in AE (42% vs 22% of cases), and thalamic and basal ganglia anomalies were also slightly more frequent. However, it must be pointed out that HSE accounted for only ~ 14% of the infectious cohort in this study. Basal ganglia involvement was also reported as a key diagnostic clue by Oyanguren et al. [100], as this sign alone had sensitivity/specificity 0.82/1 in distinguishing AE from HSE in their cohort. In addition, in their cohort insular involvement was more common in HSE. Another study [130] on 251 cases focused on distinguishing temporal lobe HSE from its mimics, including AE and other infectious/autoimmune conditions. In a multivariate model, the bilateral involvement of MTL and the presence of extra-limbic alterations were associated with a lower probability of HSE (odds ratio 0.38 and 0.37, respectively).

As for MRI signal evaluation, HSE can present with CE areas and areas of DWI restriction [133], that are rather infrequent in AE (as previously discussed). In addition, hemorrhagic spots on T2*-weighted images and necrotic areas can be seen in HSE [131, 133]. However, it is to note that the traditional necrotic-hemorrhagic appearance of HSE is seen in the late stages of the disease, and these days patients are often evaluated and imaged before these features may reveal [133].

**Fig. 6:**
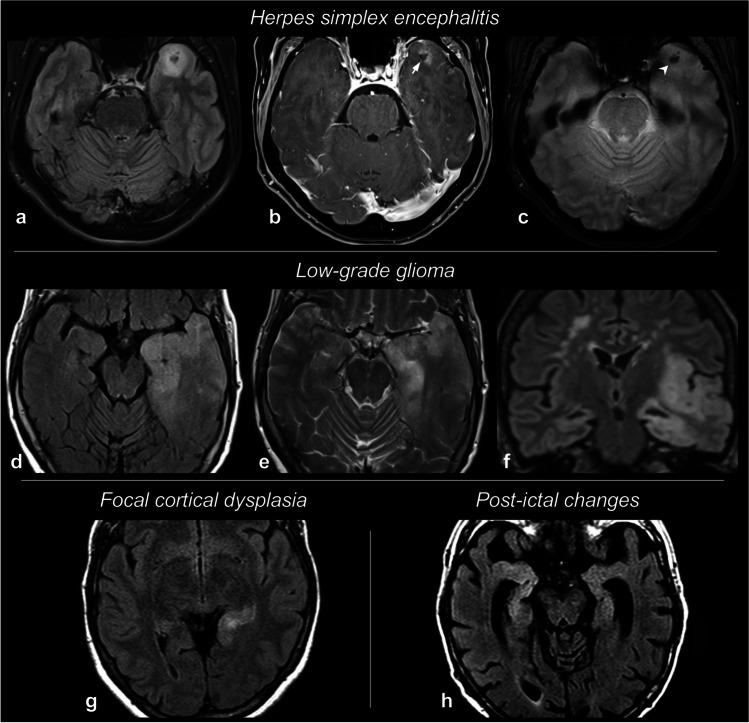
Differential diagnoses of autoimmune encephalitis. Some examples of conditions mimicking limbic autoimmune encephalitis are collected here. In a typical case of herpes simplex encephalitis (HSE) (**a**–**c**), unilateral T2-weighted FLAIR hyperintensity of the left anterior temporal areas (**a**) is associated with post-contrast T1 focal enhancement, with a central non-enhancing and hypointense spot (**b**, arrow), corresponding to hemorrhagic foci on T2*-weighted gradient-echo images (**c**, arrowhead). In a patient with low-grade glioma (LGG) (**d**–**f**), T2-weighted FLAIR (**d**, **f**) and T2-weighted TSE (**e**) exhibit a marked swelling and hyperintensity of the left anterior temporal lobe and insula, with a widespread thickening of the cortex and mass effect on the sulci. An example of focal cortical dysplasia (FCD) located in the tail of the left hippocampus is shown on T2-weighted FLAIR images (**g**). Finally, T2-weighted FLAIR images (**h**) obtained after an epileptic seizure demonstrate a MTL signal hyperintensity due to post-ictal changes

As emerges from these studies, distinguishing AE and HSE is often a challenging task, even though fever, neuropsychiatric manifestations, lesion site, and lesion signal features may point towards one or the other. In general, if HSE is suspected based on one or more characteristics, anti-viral treatment should be started immediately. Finally, it is worth mentioning that multiple pieces of evidence demonstrated that HSE itself can be a trigger for subsequently developing AE, and specifically anti-NMDAR AE [85, 134, 135]. Figure 6a–c shows a case of HSE with contrast-enhancement and hemorrhagic foci.

### Steroid-responsive encephalopathy associated with autoimmune thyroiditis (SREAT)

Previously known as Hashimoto’s encephalitis (HE), SREAT is a condition associated with autoantibodies against thyroid antigens, for which the pathogenetic role is still controversial [136]. SREAT can mimic AE both by presenting with T2/FLAIR hyperintensities in the MTL structures, and by causing memory deficits, psychiatric symptoms and seizures [12, 136]. Moreover, as seen in AE, SREAT may also be MRI-negative (~ 50% of the cases) [136]. However, SREAT is characterized by a peculiar “migratory pattern”, consisting in the disappearance of some signal abnormalities over time, while new signal alterations appear in new sites [12, 136]. Additionally, SREAT often presents a preferential white matter involvement: leukoencephalopathy with confluent T2/FLAIR alterations [12, 136]. These two MRI findings aid the distinction between SREAT and AE. In addition, approximately 1/3 of SREAT cases occur in patients with a known thyroid disfunction, according to a revision of 251 cases [136]. It is worth noting that the definition of SREAT/HE is evolving. For instance, evidence from a recent study has challenged SREAT/HE definition, by highlighting that in a cohort of patients fulfilling criteria for these conditions, these criteria are not able to actually predict response to steroids [137].

### Other infectious and immune-mediated conditions

AE with a striatal involvement, especially anti-CV2/CRMP5, may resemble other conditions affecting the basal ganglia, and particularly Creutzfeldt-Jacob disease (CJD). In the suspicion of CJD, seeking the typical patterns of DWI restriction in the basal ganglia and/or in the cortex is crucial [138].

As already discussed, some Group I AE (e.g. anti-Ma/Ta, anti-Hu) can involve the brainstem and the cerebellum. In these cases, alternative causes of rhombencephalitis should be considered, especially Listeria encephalitis. In Listeria encephalitis, enhancement is frequent and supratentorial involvement is rare. In addition, CSF examination and CSF and blood cultures may aid the diagnosis [139]. It should be noted that AE is a relatively rare cause of rhombencephalitis, as other bacterial (Listeria), viral (Herpesviridae, Epstein-Barr), and immune-mediated (Behçet) etiologies are more common [139].

Rarely, primary CNS vasculitis may resemble AE. In those cases, acquiring angiographic images to demonstrate abnormal vascular structures may be helpful. In addition, DWI is useful in case of vasculitis-induced micro-infarctions [85].

Lesions in diencephalon can also be related to neuromyelitis optica spectrum disorders (NMOSD); this involvement is characteristic but not pathognomonic for NMOSD and is considered one of features in the 2015 revised diagnostic criteria [140]. Typically, diencephalic lesions involve the peri-ependymal surfaces of the midbrain adjacent to the cerebral aqueduct and thalamus or hypothalamus adjacent to the third ventricle and the pattern of enhancement is more diffuse (cloud-like, ring-like, leptomeningeal) [28]. Other diagnostic considerations when basal ganglia are involved include sarcoidosis, IgG-4 disease-related syndromes, histiocytosis, Behçet’s Disease, Granulomatosis with Polyangiitis, ADEM, tuberculosis, fungal and bacterial infection [141].

As for the perivascular radial contrast-enhancement pattern involving the supratentorial white matter seen in anti-GFAP AE, it is worth remembering that a similar finding can be seen in neurosarcoidosis [142, 143] and sometimes in lymphomatoid granulomatosis (LYG) [144]. When this presentation is encountered, the next step in management should be dosing the serological levels of anti-GFAP to confirm or rule out anti-GFAP AE.

Finally, even COVID-19-associated encephalitis was reported to involve limbic structures (hippocampus, amygdala, MTL, cingulate gyrus) in some cases, and should be therefore be considered in the differential [145, 146], at least in SARS-CoV-2 positive patients.

### Neoplasms

Gliomas involving MTL structures can mimic limbic encephalitis, especially in cases that do not present with a striking mass effect and CE. Most of these cases are low-grade gliomas (LGG), with an infiltrative growth pattern. In these cases, typically LGG may show indistinct margins, mass effect, and extra-limbic extension (perhaps with white matter involvement), which would not be expected in AE [85]. In addition, LGG are classically unifocal and unilateral, while bilateral alterations would point to AE. However, on very rare occasions also high-grade gliomas can present with atypical imaging features and can be mistaken for LE. In a recent paper on the topic [147], the authors report that this misdiagnosis can occur in ~ 2% of suspected AE. After reviewing 13 cases of glioblastoma mimicking AE, they argue that in these cases both clinical manifestations and conventional MRI may be misleading, as patients often had memory deficits, psychiatric symptoms, and seizures, and their scans showed non-enhancing T2/FLAIR limbic alterations, bilateral in more than half of the cases. In these challenging cases, the authors recommend to perform spectroscopy and perfusion imaging, in order to detect suspicious findings advocating for glioblastoma (high Cho/Cr ratio, elevated perfusion metrics). A case of LGG mimicking LE is represented in Fig. 6d–f.

### Seizure-related alterations

Seizure-induced T2/FLAIR alterations, related to post-ictal edematous changes, may resemble LE, too [85]. Recently, Budhram et al. [111] suggested that in the presence of specific hippocampal diffusion restriction patterns (gyriform and/or diffuse) the diagnosis of post-ictal changes should be favored, as opposed to AE. ADC reduction following seizures are considered the result of post-ictal cytotoxic edema. Follow-up imaging, as well as electrophysiology studies, may also be useful to differentiate these two conditions. Figure 6h shows a case of post-ictal MTL alterations.

## Suggested imaging diagnostic work-up

When clinical history and neurological and electrophysiologic (i.e. EEG) examination evoke the suspicion of AE an extensive workup is necessary to confirm the diagnosis and to exclude other pathological entities as reported in the previous chapters.

### Neuroimaging

Neuroimaging plays an important role in the work up. Brain CT is frequently the first imaging modality used, particularly with subacute presentations when the patients are admitted in the ER. It must be stressed that CT is not sensitive for the identification of brain abnormalities in AE and MRI should be always acquired whenever possible. In fact, brain MRI is key in identifying the presence of brain abnormalities in AE and to characterize the anatomical pattern of involvement (i.e. limbic or extra-limbic). It is worth noting that in the acute phase patients can be uncooperative and MRI under sedation could be required.

MRI should be acquired with and without gadolinium-based contrast agents and the acquisition protocol ideally should include high resolution 3D-T1, T2-FLAIR, TSE T2, DWI, SWI and post-contrast T1 [148–150]. 3D T1-weighted images provide more anatomical detail to identify enhancing areas, and also are helpful to monitor atrophic changes over time. 3D turbo spin-echo (TSE) is superior to 3D inversion-recovery gradient-recalled echo (IR-GRE) in the identification of small foci of contrast-enhancement (higher lesion conspicuity), as proven on brain metastasis studies [151, 152]. If IR-GRE is used, it may be advisable to also obtain additional 2D spin-echo T1 images after contrast, which may improve the detection of small enhancing foci, as already recommended for the identification of small brain metastases [151, 152]. As for T2-weighted and/or T2-weighted FLAIR images, it is advisable to acquire also coronal images to evaluate the volumes and symmetry of medial temporal lobes structures. As for DWI, classic clinical sequences with *b* = 1000 and ≥ 3 directions are generally sufficient. Recent case series suggest the role of perfusion studies, including arterial spin labeling (ASL) in the pediatric population [122].

Ideally, brain MRI should be obtained before the lumbar puncture to avoid difficulties in the interpretation of post contrast imaging, with pachymeningeal thickening and enhancement, frequently detected in the setting of post lumbar puncture CSF hypotension. However, when encephalitis is suspected, guidelines recommend timely collection of CSF in order to assess viral etiologies and start empiric antiviral treatment. CSF collection in encephalitis is also crucial to test for anti-neuronal antibodies, that are pivotal to diagnose AE. The appropriate timing of brain MRI should be evaluated in each patient, carefully considering the likelihood of an underlying viral etiology that would prioritize lumbar puncture over brain imaging [153]. Brain FDG-PET also plays an important role since it can provide additional information of brain involvement also in patients with negative MRI. Specific metabolic patterns were shown to correlate with antibody types – e.g., occipito-parietal hypometabolism axnd anti-NMDAR, MTL hypometabolism with anti-LGI1 and with onconeural antibodies [154].

### Whole body imaging

Computed tomography (CT) of the chest, abdomen, and pelvis with contrast is recommended as the first screening for associated malignancies in those cases where a paraneoplastic etiology is suspected. Importantly, the type of neuronal antibody detected is crucial to stratify cancer risk and to orient towards specific cancer subtypes. Additionally, some clinical phenotypes are also associated with a higher risk for accompanying cancer [17].

The main limitation of the CT evaluation is its low sensitivity to early breast and testicular cancers; if these tumors are suspected a mammogram or testicular ultrasound should be performed [155]. Breast MRI should then be obtained if mammography is negative but breast cancer suspicion remains high. Young male patients (< 50 years) with a diagnosis of anti-Ma2 AE may have microscopic testicular neoplasms even in the absence of positive ultrasound findings [156]. In case of a negative testicular ultrasound, suspicious clinical signs (e.g., testicular enlargement) and risk factors (e.g., cryptorchidism) should be investigated, and these patients should be closely monitored with repeat ultrasound in order to identify the potential appearance of microcalcifications at later follow-ups [156].

Transvaginal ultrasound or pelvic MRI to assess the presence of ovarian teratoma or adenocarcinoma must be considered in female patients, especially in the case of young and middle-aged women with anti-NMDAR encephalitis.

Whole body FDG-PET has a higher sensitivity when compared to CT for the detection of occult neoplasms, and should be obtained if the initial screening is negative and the suspicion of cancer is high, or in patients with carcinoma unknown primary (CUP) syndrome.

In patients with paraneoplastic neurological syndromes (PNS) and paraneoplastic antibodies if primary screening is negative, it is recommended to repeat screening after 3–6 months and then screen every 6 months up till 4 years [155].

## Conclusions

**Fig. 7:**
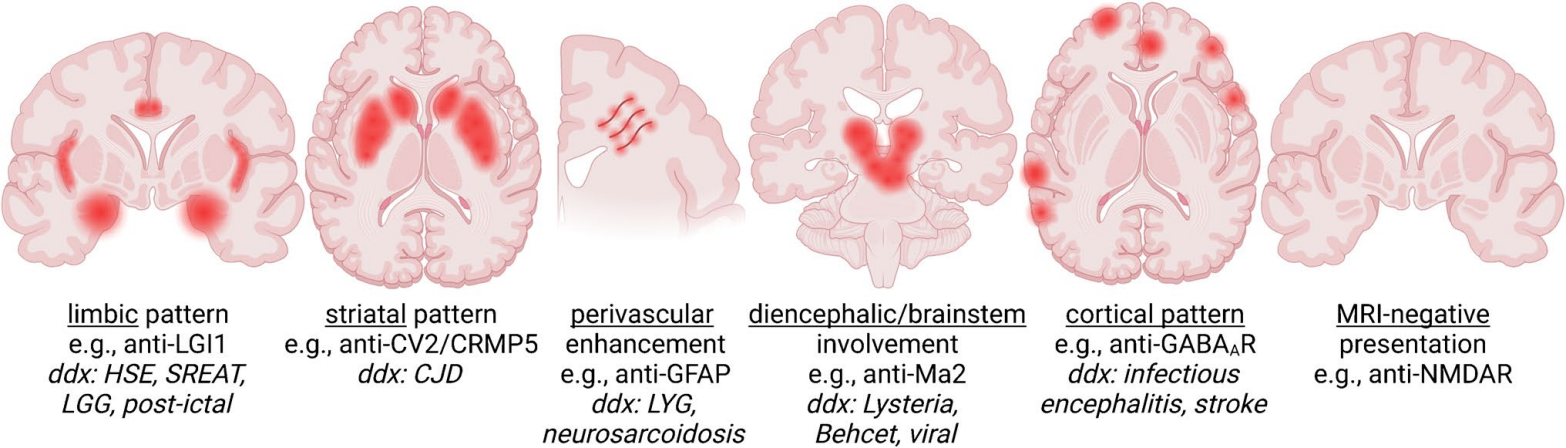
Schematic overview of the main radiographic patterns in AE, along with examples of associated autoantibodies and with the main differential diagnoses. Note that more than one presentation can be seen for each autoantibody, and that sometimes more than one pattern can coexist (e.g., limbic plus brainstem) HSE = Herpes simplex encephalitis, SREAT = steroid-responsive encephalopathy associated with autoimmune thyroiditis, LGG = low-grade glioma, CJD = Creutzfeldt-Jakob Disease, LYG = lymphomatoid granulomatosis. Created with BioRender.com

Autoimmune encephalitis (AE) is a challenging diagnosis, as it includes a variety of subtypes that differ in clinical presentation, radiologic appearance, and serologic findings. Additionally, it is often misdiagnosed, since it can mimic other conditions. The radiologist should be aware of the main imaging findings associated with these entities, including limbic and extra-limbic patterns (Fig. 7), as well as the useful clues for differential diagnosis, and an overall understanding of the clinical and biological underpinnings of this disease. A prompt diagnosis of AE can improve the prognosis both by expediting immunosuppressive treatment and allowing for the screening for an underlying malignancy.

## Abbreviations

AE: Autoimmune encephalitis
AMPAR: α-Amino-3-hydroxy-5-methyl-4-isoxazolepropionic acid receptor
CASPR2: Contactin-associated protein-like 2
CRMP5: Collapsin response mediator protein 5
D2R: Dopamine receptor 2
GABAR: Gamma-Amino butyric acid receptor
GAD: Glutamic acid decarboxylase
GFAP: Glial fibrillary acidic protein
Gluk2: Glutamate kainate receptor 2
HSE: Herpes Simplex Encephalitis
KLHL11: Kelch-like protein 11
LE: Limbic encephalitis
LGI1: Leucine-rich glioma inactivated
MOG: Myelin oligodendrocyte glycoprotein
NMDAR: N-Methyl D-Aspartate Receptor
PCA2: Purkinje Cell Cytoplasmic Ab Type 2
PERM: Progressive encephalomyelitis with rigidity and myoclonus
SPS: Stiff person syndrome
VGCC: Voltage gated calcium channel

## Declarations

### Editorial Board Members and Editors

The authors have no editorial positions in this journal.

### Employment

The authors have no relevant employment to disclose.
